# Editorial

**Published:** 1860-06

**Authors:** 


### Editorial.

The boy that encountered and killed the skunk, explained to his comrades that he would have kept out of the fight if he had known the thing *wasn't a cat;* but as he had to endure the stink, he thought he might as well have the victory. Like him, we have got into a controversy by being mistaken in the nature of the animal; and being deceived into it, like him, we conclude to go through with it.

We have before referred to a couple of articles, written by an anonymous contributor, and published in the American Journal, one of them on Dental Periodicals, and the other on Dental Societies. We thought these articles did injustice to our profession, and presuming that they were written by a member of the same, we noticed them as we thought they deserved. We, also, gave a description of the mental condition of a dentist who could write such stuff. In the April number of the Journal the writer, to show that our description of him is inaccurate, says: "Nor have we ever been of the *profession,* or of the *auxiliary* branches of science, as understood by the American Dental Convention."

Now, the boy, in the case referred to, could not blame the skunk for deceiving him; for, though it looked something like a cat, it didn't profess to be one. But this writer in both his former articles, professes to be a member of the Dental Profession, and now, when cornered, confesses himself an *outsider.* True, he says: "I inadvertantly made use of the word *our* in connection with the profession in some places." Most of his readers will conclude that he was persistently, at least, if not impertinently inadvertent. Some will admit his pleas, and some will think he used the term on purpose to deceive When the jackdaw decked himself in stolen plumage, and went into peacock society, why did he not set up the plea of *inadvertance,* when about to be packed out for his impudence?

Just think of it, reader. Here is a man who has never "been of the profession," "never been present at any meeting of any Dental Society whatever," and yet, in one of our journals, he denounces our periodicals, condemns our associations, ridicules the greater part of the profession, and *lectures* the remainder, criticises and burlesques our style, (in miserable English at that), intimates that our endeavors after improvement are contemptible efforts, in short, does everything that a *modest* man would not do, and all this while *pretending* to be one of our number. How kind it was in him, after informing us that all our societies—local as well as national—have proved worse than failures, to tell us just how to get one up! As kind as the owl that, without invitation, gave free lectures on music, to a class of thrushes and nightingales; and as consistent and reasonable as the *ass* that told the horses that if their ears were larger, their tails less bushy, their eyes less bright, and their necks less arched, they would make a much more respectable appearance.

Since we have undisguised our man, we have no disposition to continue a controversy with him, no desire to shoot the last, or even any arrow; for we agree with him that a man who will assume a *false character* that he may the more effectually impugn the motives and actions of others, is too small a mark for our aim. We suppose this is his idea when he says of us: "He shall enjoy his apparently much prized practice of shooting the last arrow, for his aim is far too inaccurate, and his *mark too small* for us to feel the least concern." The italics are ours.

The late article of this contributor is entitled "Corrections." He might correct some *few* errors of his own. We never intimated that the "Mississippi Valley Association" was other than a local society. On the contrary, we referred to it to prove that local societies are not all failures. Again, we never abandoned our gold for our pen; and if we did, whose business is it! But he is excusable for these, and for many other mistakes, as he is yet laboring under his "mathematical hallucination," as all who understand the English language can readily see, by comparing his last two articles with each other.

In reference to one of our remarks on his former articles, this writer says: "We are correct in thinking that the most saucy Grube Street author would not have assailed us with such a miserable quibble as this," which may be all true. We are not acquainted with the authors on that street; and we make it a rule not to dispute with a man, when we are not in possession of the facts and he is. We take it for granted that our friend has a thorough acquaintance with these authors; for if not, how could he affirm so positively that he is *correct* in his conclusions as to what they would or would not do?

Near the conclusion of his article, in speaking of us, he says: "The fertility of his resources at length appear to be exhausted,—for, far from seizing our evident meaning, he presumes to intimate," etc. He is responding, here, to the concluding part of our former article; and at this point, or "at length," he suspects that the fertility of our resources is exhausted, because we are "far from seizing" his evident meaning, which amounts to a pretty plain intimation that before this exhaustion took place, we were not "far from seizing" his evident meaning. Hence, all his previous efforts at making "corrections," are, by his own statement, lost labor.

He does not like our criticism on his "outline" of a plan of organization for a Dental Society. He says: "We should remember that, among intelligent men, special provisions is always made for the reception of the words of the wise." We did remember this, and taking it for granted that he was an intelligent man, were surprised that he had made no such special provision in his outline.

But it is time to close. We are perfectly willing to be advised in regard to Dental Periodicals and Societies, by tinkers, cobblers, farmers, mechanics, physicians, lawyers, preachers or philosophers, provided they sail under their own colors; but we are not willing that pirates should profane *our* flag. W.

### CHEMICAL ANALYSIS.

In the May number of the "American Dental Review" there is a curious editorial on "OS-ARTIFICIAL." We have nothing to say of the *gross* misrepresentations contained in the first paragraph; for our comrade is able to take care of himself. Were it not for a single sentence in the second paragraph, we would not have had any thing to say.

Roberts' "Os-artificial" being under consideration, the "A. B." editor says, "I ask—why has not the Professor of Chemistry analyzed it, and published the results to the world? It is the universal practice among the Universities of Europe to take up and analyze every new patent medicine or secret nostrum—."

His autocratic "*I ask*," would lead Mrs. Partington to think he was the "Professor over all the Faculties." It may be as he states among the universities of Europe; but the chemists are as universally paid for their labors. We dislike the tone in which the question is asked. He appears to think he has a right to demand the special labors of the professor of chemisty. But we will *imagine* that the question is put with all due respect, and that the writer wishes a candid answer. We would state, then, that the "professor of chemistry" is already weighed down with more important chemical researches, that, for the last seven years, he has *given* more mental labor to the profession than he has devoted to his own interests, that he has had but two days of mental relaxation in these seven years, and that he has been so foolish as to suppose he would receive the sympathy of those for whom he labored, instead of an arrogant demand for more toil. This last we know is a humiliating confession of weakness.

In conclusion, *I ask*—why has not "A. B." hired some good chemist of his vicinity to analyze it, and publish the results to the world? As he is so very anxious about it, we wonld inform him that the way is still open. He can as well afford to pay a neighbor for doing it, as we can afford *our* time to do it. W.

### A CORRECTION.

WE observe in the May number of the "American Dental Review" some editorial remarks upon *Os Artificial*, in which the writer makes some strictures upon our remarks made in the April number of the Dental Register on the same subject.

On this subject we should have nothing further to say, had we not been grossly misrepresented by A. B., and things attributed to us we did not say, and this designedly, for he evidently had our language before him.

He says: "He tells us that it is *invaluable* for building crowns of teeth which are partially broken away, and that it *will* last from two to five years."

Now, what we did say is this: "Again, it is valuable for filling teeth that are very much decayed, and for building up crowns that are partially broken away." "It is valuable in this respect only for molars and bicuspids." Now, the only difference between A. B.'s quotation and the truth, is the difference between *invaluable* and valuable. *Invaluable*—precious above estimation ; so valuable that its worth can not be estimated. *Valuable*—having value or worth ; having some good qualities, which are useful and esteemed.

In regard to the time which it will last, we are represented as saying that it *will* last from two to five years. The following is what we did say, viz : " Of course, it will wear down with more or less rapidity, according to the force with which the teeth are used, but it *seems* to be sufficiently firm to wear for a considerable time, *probably* from two to five years."

Most persons would conclude that there was some qualification in regard to the durability of this material, as expressed above ; but of course some allowance should be made for difference of opinion. We would suggest that A. B. be a little more careful in his quotations in the future, especially if he intends to maintain a reputation for veracity.

In regard to what we said of *os* artificial, we have as yet not one word to retract. With this we did just what we do in all such cases—speak just what we think at the time, according to the light we may have.

When we deem it important to direct the attention of the profession to any particular matter, we aim to present the merits and demerits, so that those who desire to know, may arrive at a tangible conclusion.

We were not investigating its composition—we supposed every one knew as much about that as it is important to know. Various formulas of a similar character have been published in almost all the journals during the last year. T.

The italics in the above quotations are our own.—Ed.

---

*Chemistry for Farmers—The elements of Chemistry as applied to Agriculture. By C. B. Chapman, A. M. M. D., Professor of Chemistry.*

This is a work gotten up for farmers and schools. It is the application of Chemistry to agriculture, a very important subject. It alone can reveal what is all important to be known to the agriculturalist; without it he labors in the dark. The work to which we refer is designed to give the needed information upon all the important particulars connected with this matter. The work embraces nineteen chapters, in which the subjects are naturally and most conveniently arranged. A clear and concise consideration of the elements is first given. Those elements that enter into the composition of soil and plants are more prominently set forth, in their combinations and mixtures. The subjects of manures, their natures, varieties, and application are clearly discussed. Every one who takes any interest in the subject of agriculture (and who should not) cannot fail to be interested and instructed by a perusal of this little work, but to the farmer it will be almost invaluable, as he will find there just what he wishes to know, without any circumlocution, or redundancy of language, expressed in language he can understand. It is published by W. B. Smith & Co., of this city, and may be obtained wherever their school books are for sale. T.

---

We copy the following from the Vulcanite. We can only say now that a death like this brings sorrow to every one acquainted with the subject of it.—Ed.

"It becomes our painful duty to record the death of Dr. Alvan Blakesly, of Utica, N. Y. The circumstances attending his death are of the most melancholy character. As near as we are able to learn, Dr. Blakesly left Utica about the middle of last month to recuperate his health, his system having become debilitated by constant application to business. His physician and numerous friends advised him to go South for a short time, in order, if possible, to regain his health. He stopped at a friend's a short time in this city, expressing a desire that no one should know of his being here, giving his reason that he was too unwell to see them. He took passage in one of the Savannah steamers, in company with an acquaintance of his from Utica; and, on or before their arrival at Savannah, Dr. Blakesly was missing, it being believed that he had fallen overboard and was drowned. The circumstances connected with his mysterious disappearance from the decks of that steamer may forever remain a mystery to man; but his many virtues, his companionable and gentlemanly deportment, and his efforts to elevate his profession by friendly intercourse, will be remembered by many who will ever cherish for his name the fondest recollections."

